# Profiling Invasiveness in Head and Neck Cancer: Recent Contributions of Genomic and Transcriptomic Approaches

**DOI:** 10.3390/cancers7020585

**Published:** 2015-03-31

**Authors:** Lluís Nisa, Daniel Matthias Aebersold, Roland Giger, Marco Domenico Caversaccio, Urs Borner, Michaela Medová, Yitzhak Zimmer

**Affiliations:** 1 Department of Radiation Oncology, Inselspital, Bern University Hospital, and University of Bern, Bern 3010, Switzerland; E-Mails: daniel.aebersold@insel.ch (D.M.A); michaela.medova@dkf.unibe.ch (M.M.); 2 Department of Clinical Research, Inselspital, Bern University Hospital, and University of Bern, MEM-E807, Murtenstrasse 35, Bern 3010, Switzerland; 3 Department of Otorhinolaryngology—Head and Neck Surgery, Inselspital, Bern University Hospital, and University of Bern, Bern 3010, Switzerland; E-Mails: roland.giger@insel.ch (R.G.); marco.caversaccio@insel.ch (M.D.C.); urs.borner@insel.ch (U.B.)

**Keywords:** head and neck squamous cell carcinoma, cell invasion, whole exome sequencing, gene expression profiles, *NOTCH 1*, *PI3K*, epithelial-to-mesenchymal transition

## Abstract

High-throughput molecular profiling approaches have emerged as precious research tools in the field of head and neck translational oncology. Such approaches have identified and/or confirmed the role of several genes or pathways in the acquisition/maintenance of an invasive phenotype and the execution of cellular programs related to cell invasion. Recently published new-generation sequencing studies in head and neck squamous cell carcinoma (HNSCC) have unveiled prominent roles in carcinogenesis and cell invasion of mutations involving *NOTCH1* and PI3K-patwhay components. Gene-expression profiling studies combined with systems biology approaches have allowed identifying and gaining further mechanistic understanding into pathways commonly enriched in invasive HNSCC. These pathways include antigen-presenting and leucocyte adhesion molecules, as well as genes involved in cell-extracellular matrix interactions. Here we review the major insights into invasiveness in head and neck cancer provided by high-throughput molecular profiling approaches.

## 1. Introduction

Head and neck squamous cell carcinoma (HNSCC) is the 6th most prevalent type of cancer and arises in the mucosa of the upper aerodigestive tract. HNSCC presents at an advanced stage in 40%–60% of the cases, most often requiring multimodal treatment with surgery and radiation (+/− chemotherapy) [1,2]. Despite the implementation and improvement of such multimodal regimens over the last few decades and the recent FDA approval of the anti-epidermal growth factor receptor (EGFR) antibody cetuximab in combination with radiotherapy, up to 50% of patients still experience local and/or regional recurrence, or develop distant metastases. Unfortunately, the prognosis of recurrent HNSCC is most often dismal [1,3,4].

HNSCCs are primarily tobacco-related neoplasms, but infection by high-risk subtypes of human papillomavirus (HPV) has also been established as an important etiologic factor that accounts for a trend for increasing incidence of oropharyngeal cancers in men younger than age 50 years without a history of tobacco use [1,5,6]. HNSCCs tend to metastasize to regional lymph nodes early in disease progression. The presence of lymph node metastases is the most important prognostic factor identified so far in HNSCC [1]. Distant metastases usually occur later in progression, often after definitive therapy has been delivered to the primary tumor and the regional lymph nodes [7].

Recent DNA and RNA profiling studies in HNSCC indicate a high level of underlying molecular heterogeneity [8]. The evolving genomic and transcriptomic technologies combined with large-scale integrative tools of systems biology have emerged as powerful methods to approach such a complex disease [8,9,10]. From a translational perspective, understanding specific mechanisms of invasion may allow tailoring more patient and tumor-specific management strategies [11].

**Figure 1:**
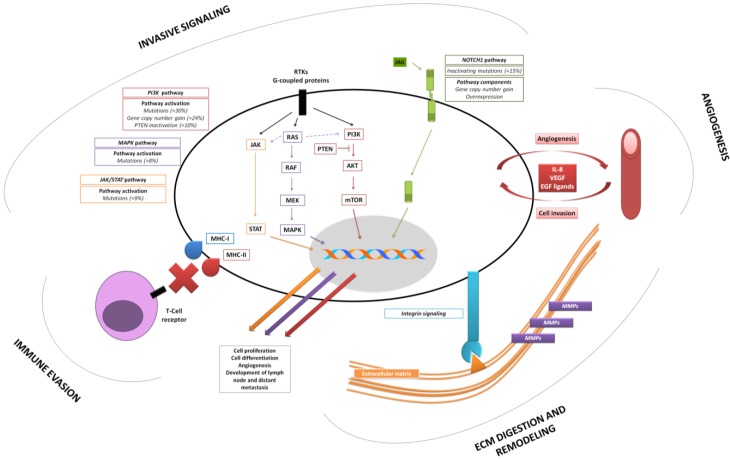
Most relevant mechanisms of cell invasion outlined by high-throughput profiling studies in head and neck squamous cell carcinoma (HNSCC).

In this review we discuss some of the most relevant and illustrative insights outlined from recent genomic and transcriptomic approaches in relation with tumor invasiveness in HNSCC (Figure 1).

## 2. The Profile of the Invasive HNSCC

### 2.1. Recent Genomic and Transcriptomic Findings—Impact on Invasiveness of HPV-driven HNSCCs

Comprehensive genomic and transcriptomic data has been recently published by The Cancer Genome Atlas Network [12]. The findings of this study, along with the data previously reported by Seiwert *et al.* [13], provided important insights into the similarities and differences between HPV negative (tobacco-driven) *vs.* HPV positive tumors. In broad terms, HPV negative tumors were similar to lung and esophageal squamous cell carcinomas with respect to mutational profiles, characterized by activating alterations of receptors tyrosine kinase (RTKs)-RAS-PI3K pathways, as well as mutational inactivation of *TP53* and *CDKN2A*. In contrast, HPV positive tumors were characterized by activating alterations of *PIK3CA*, *FGFR3*, and *E2F1* along with inactivation of *TP53* and *RB1* by the viral oncoproteins E6 and E7 respectively [12,13]. Furthermore, HPV positive tumors showed less chromosomal aberrations than HPV negative tumors, but both groups shared common features. For instance, the presence of recurrent amplifications of the 3q26/28 region was common in HPV− and HPV+ tumors. This region contains essential genes involved in squamous lineage (e.g., *TP63*, *SOX2*), as well as *PIK3CA* (encoding the p110α subunit of PI3K, discussed in Section 2.4) [12]. Importantly, activating mutations of *PIK3CA* are significantly more prevalent in HPV+ tumors [14].

A very relevant finding in HPV+ tumors was the discovery of previously unknown recurrent deletions and truncating mutations of TNF receptor-associated factor 3 (*TRAF3*), a gene implicated in anti-viral responses whose loss leads to aberrant activation of NF-κB (nuclear factor-κB) [15,16,17]. In turn, NF-κB is a master modulator of the inflammatory response and has been clearly linked to oncogenesis and disease progression in several types of cancer [18].

While these observations underline basic differences in oncogenesis in HPV− *vs.* HPV+ tumors, further work is needed to fully characterize HPV-related mechanisms of invasion in HNSCCs.

### 2.2. Acquiring an Invasive Phenotype—Relevance of Cell Differentiation in HNSCCs

Several prerequisites must be met for a highly differentiated, polarized and contact-dependent epithelial cell to become invasive. Basically, cells must acquire resistance to apoptosis-anoikis, ability to digest and remodel the extracellular matrix (ECM), become motile, and develop immune evasion. Additionally, epithelial cells must be able to survive in hostile environments such as lymphatic or blood vessels [19,20,21].

Epithelial cells can acquire mesenchymal-like features through epithelial-to-mesenchymal transition (EMT) [20,22]. Even though EMT is a plausible model that accounts for many features of invasive cells, acquisition of a mesenchymal phenotype is not compulsory for HNSCCs to invade [22,23,24].

In line with this, in a gene expression profiling study of 60 HNSCCs, Chung *et al.* [25] were able to clearly delineate two subgroups of tumors based on their epithelial *vs.* mesenchymal differentiation. Immunohistochemical analysis of tumors in the epithelial subgroup revealed expression of different members of the cytokeratin family, while mesenchymal tumors were characterized by high expression levels of vimentin, abundance of stromal fibroblasts, and regions of desmoplastic reaction (potentially suggesting active EMT). From a clinical perspective, patients with mesenchymal tumors had a decreased recurrence-free survival [25]. An ulterior gene expression profiling study by Chung *et al.* [26] supported these observations and revealed that expression of genes involved in EMT, cell adhesion, and nuclear factor-κB signaling defined a high-risk subtype of HNSCCs. The findings of these studies and several others suggest that different phenotypes within the epithelial-to-mesenchymal spectrum may imply different mechanisms of invasion in HNSCC [27,28,29].

### 2.3. NOTCH1 Functional Duality as an Emergent Link between Initiation and Invasion of HNSCC

Two independent next-generation sequencing (NGS) approaches were published in 2011, further emphasizing the relevance of cell differentiation in tumoral invasion and providing new insight into mechanisms of oncogenesis and invasion. Stransky and colleagues [30] processed data from 74 and Agrawal *et al.* [31] from 32 HNSCCs. The most relevant feature in both studies was the discovery of an excess of mutations in a set of genes functionally related to epithelial squamous differentiation. Most specifically, these mutations concerned the *NOTCH1* gene. This gene emerged as the second most common alteration in HNSCC (mutated in approximately 15% of the cases) [30,31].

The gene product of *NOTCH1* is a transmembrane receptor whose intracellular domain translocates to the nucleus and acts as a transcription factor upon binding of extracellular ligands [32]. Activation of Notch signaling has crucial roles in embryogenesis, cell differentiation (partly through regulation of EMT), angiogenesis, resistance to anoikis, and in the context of cancer, development of metastases [33,34,35,36]. Initially *NOTCH1* was considered a paradigmatic oncogene due to its aberrant signaling in certain hematopoietic malignancies [32]. Nonetheless, this notion was very quickly challenged following contradicting observations in solid tumors, in which *NOTCH1* seemed to act as a tumor-suppressor [37].

Recent genomic studies in HNSCC suggest the co-existence of a tumor-suppressing and an oncogenic role for Notch signaling. Indeed, on one hand most *NOTCH1* mutations found in the recent NGS approaches in HNSCC were predicted to result in loss of function and therefore point towards a tumor-suppressive role [30,31]. This observation is fully congruent with the increased rate of cutaneous SCCs reported both in *NOTCH1* knockout murine models and in patients enrolled in an early clinical trial testing a Notch signaling inhibitor (gamma secretase inhibitor) [38]. On the other hand, recent evidence shows activation of the Notch pathway in HNSCC as an oncogenic mechanism. Sun *et al.* [39] assessed gene copy number variation, promoter methylation status, mutation, and expression of several members of the Notch signaling pathway in a cohort of 44 tumors and 25 matched normal mucosa specimens. Remarkably, eight components of the Notch signaling pathway, including Notch ligand JAG1, displayed significant gene copy number gain in tumors. In line with this, mRNA levels of 15 components of the Notch pathway as well as their transcriptional targets were significantly up-regulated. These findings strongly suggest an oncogenic role of Notch signaling [39]. The interactions between Notch and other essential players in cell invasiveness transcend the Notch pathway. An illustrative example of this in the recent TCGA data is the co-existence of *FAT1* inactivating mutations and Notch alterations. Active *FAT1* sequesters β-catenin, resulting in inhibition of Wnt-signaling [12]. These findings confirm previous studies and add mechanistic insights into the essential interplay between Notch and Wnt signaling [40].

Taken together, these recent results indicate that in certain subsets of HNSCCs, loss of *NOTCH1* function as tumor-suppressor contributes to oncogenesis and invasiveness, while in another subset of tumors, Notch signaling pathway activation may fuel cell invasion through EMT, induction of angiogenesis, and resistance to cell death [30,35,39,41]. The factors that condition whether Notch acts as a tumor-suppressor or as an oncogene are not fully understood. Some evidence suggests that tissue type and even tumoral stage may play a role in determining the outcome of Notch dual role. Finally, it is important to point out that Notch functional duality poses an obvious challenge to effectively implement therapies targeting Notch signaling [42].

### 2.4. New Insights into Old Players: Implications of the Mutational Landscape of Mitogenic Pathways

Several hallmarks of cancer such as sustaining proliferative signaling, activating metastasis, and angiogenesis are partially executed through aberrant activation of receptors tyrosine kinase (RTKs) and G-coupled proteins following receptor/ligand overexpression and activating mutations [43]. Additionally, aberrant signaling can also occur due to primary activation of one or more components in certain canonical pathways, primarily the mitogen-activated protein kinase (MAPK) pathway, the phosphatydilinositol-3-kinase (PI3K) pathway, and the Janus kinase-signal transducer and activator of transcription (JAK-STAT) pathway [44]. Important cross-talk exists between the MAPK and the PI3K pathways, as well as between MAPK and the JAK-STAT pathways. Such cross-talks enhances invasiveness and represents a potential mechanism of resistance to molecular targeted therapy [45].

Using data from the NGS studies described in the previous chapter along with additional data from 45 HNSCC samples, Liu *et al.* [46] focused on mutations affecting the MAPK, PI3K, and JAK-STAT pathways in a total of 151 tumors. Their findings regarding the mutation rate in components of the MAPK and JAK-STAT pathways were consistent with previous reports (8% and 9.3% respectively), but the authors reported an unexpectedly high mutation rate of PI3K signaling pathway components of 30.5% [46]. These mutations primarily affected the catalytic and the regulatory helical domains of the p110α subunit of PI3K (*PIK3CA*). Most of the mutations reported, some of which were previously unknown, conferred constitutive PI3K activation and resulted in enhanced *in vitro* growth [46]. Additionally, inactivating mutations of phosphatase and tensin homologue (*PTEN*), which antagonizes PI3K signaling, were reported in around 10% of HNSCCs [46]. Mutations in other components of the pathway (e.g., *AKT2*, *PIK3R1*, and *MTOR*) were seen in less than 4% of the cases, but tumors containing mutations affecting diverse pathway components were not uncommon, especially in advanced-stage HNSCC [46]. Furthermore, *PIK3CA* gene amplification was reported in 24.4% of tumors [46].

In conclusion, multiple mechanisms of PI3K aberrant activation seem to exist in HNSCC (Figure 1), conferring increased survival, proliferation, motility, extracellular matrix (ECM) digestion, and angiogenesis [47]. Activating PI3K mutations ultimately results in resistance to apoptosis as well as in development of metastases in preclinical models [48,49,50]. As a consequence, several therapeutic strategies targeting aberrant PI3K signaling are being explored in HNSCC [46,51,52].

### 2.5. Execution of Tumor Cell Invasion—Basic Features Underlined by Gene-Expression Profiling Studies

A number of studies have assessed gene expression profiles of HNSCCs in the last decade, providing a rather accurate and consistent picture of the key players involved in the invasive process in HNSCC (Figure 1). Table 1 summarizes some of the key processes and molecules identified and/or confirmed as essential in invasion and progression of HNSCC. As can be seen, invasive HNSCCs display up-regulation of genes related to ECM remodeling and digestion, chemotaxis, and angiogenesis. Additionally, in 2008 Yu *et al.* [53] reported the results of a network-based meta-analysis with data from 63 published works. The authors specifically sought to identify altered gene expression patterns acquired during progression from premalignant lesions, through invasive primary tumors, and lymph node metastases. The majority of enriched pathways in the meta-analysis were related to tumor-stroma interactions, such as antigen presentation, chemokine signaling, integrin signaling, leucocyte extravasation, tight junction regulation, and vascular endothelial growth factor (VEGF) signaling [53].

Remarkably, many of the molecules in Table 1 play roles in several biological processes involved in invasion. For instance, interleukin (IL)-8, initially described as a chemotactic molecule for leucocytes, has been shown to act as mitogen, motogen, and pro-angiogenic factor [54]. With respect to angiogenesis, IL-8 receptors are expressed both by tumor and endothelial cells in HNSCC, suggesting the existence of a paracrine loop: Tumor cells stimulate angiogenesis while endothelial cells in turn stimulate tumor cell proliferation and invasiveness (Figure 1). Further emphasizing the functional overlap of several molecules listed in Table 1, RTKs and their ligands, such as EGFR, MET, and fibroblast growth factor receptor (FGFR), are often overexpressed in HNSCC, and have pleiotropic effects in tumor cells, including cell differentiation, proliferation, motility, and angiogenesis [55,56,57,58].

Among the genes consistently up-regulated across different studies (Table 1) are enzymes involved in digestion and remodeling of the ECM, such as matrix metalloproteinases (MMPs) or urokinase plasminogen activator (uPA) (Table 1). MMPs can be secreted by both neoplastic and stromal cells, and are designed to digest certain components of the ECM [59]. MMPs are aberrantly expressed at very early stages of tumorigenesis and are essential in order to promote and allow cell migration and ultimately metastases [60,61]. It is important to point out that MMPs are often expressed as enzymatically inactive pro-MMPs. Therefore, not only overexpression of MMPs but also mechanisms of activation such as protein-protein interactions have major implications in cancer progression and invasion. For instance, activation and activity of MMP-9, one of the most widely overexpressed MMP in different types of cancers is enhanced by NGAL (neutrophil gelatinase-associated lipocalin), which additionally protects MMP-9 from degradation [62]. Expression profiles of the pro-MMP-9/NGAL complex has been suggested as a potential prognostic marker [63].

Finally, as an essential premise to invasion, tumor cells must be able to evade the host immune response [64]. In line with this, in the meta-analysis by Yu *et al.* [53] down-regulation of major histocompatibility complexes (MHCs) I and II was a hallmark of invasive HNSCCs.

**Table 1:** Summary of illustrative findings related to invasiveness from gene-expression profiling studies in HNSCC.

Reference(s)	Function	Sense of Regulation in HNSCC	Gene(s)
Ye *et al.* [65]; Nagata *et al.* [66]; Kainuma *et al.* [67]; Kondoh *et al.* [68]; Choi and Chen [69]	Digestion and remodeling of ECM	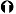	*MMP-1*
			*MMP-3*
			*MMP-9*
			*MMP-10*
			*MMP-13*
			*MMP-2*
			*uPA*
			*ITGA3*
			*ITGA5*
Ye *et al.* [65]; Gottschlich *et al.* [70]	Chemotaxis Lymphocyte activation	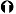	*IL-8*
			*CXCL1*
			*CD28*
			*CD3D*
			*CD4*
			*IL-18*
			*IL-2*
Kondoh *et al.* [68]; Yu *et al.* [53]	Antigen presentation	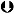	*MHC-I*
			*MHC-II*
Gottschlich *et al.* [70]; Yu *et al.* [71]	Angiogenesis	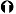	*VEGF signaling*
			*IL-8*
Gottschlich *et al.* [70]; Sun *et al.* [39]	Signal transduction	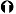	*EGFR*
			*STAT-3*
			*PI3K*
			*NOTCH*

Abbreviations: 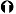
*—*Up-regulation; 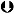
*—*Down-regulation; *MMP—*Matrix metalloproteinase; *ITGA—*Integrin alpha; *MHC—*Major histocompatibility complex; *VEGF—*Vascular endothelial growth factor; *IL-8—*Interleukin-8; *CXCL1—*Chemokine (C-X-C motif) ligand 1.

### 2.6. Paving the Tracks of Invasion—Relevance of Integrin Signaling

The most prominent finding in the meta-analysis of Yu *et al.* [53] was probably related to changes of expression patterns in different members of the integrin family. Integrins are transmembrane receptors that play important roles in cell-cell adhesions and cell-ECM interactions, and regulate cell growth, differentiation, and apoptosis [71]. The results reported by Yu *et al.* [53] are fully in line with previous *in vitro* observations by Gaggioli *et al.* [72]. The latter used a complex organotypic co-culture system, employing the facial cancer cell line SCC-12 (which retains expression of markers of epithelial differentiation), fibroblasts derived from an oral tumor, and an artificial matrix. The authors showed that SCC-12 cells tended to invade in compact groups, and in the presence of stromal fibroblasts, the leading cell of such groups was always a fibroblast [72]. To further assess the role of fibroblasts in leading invasion of epithelial cells, fibroblasts were cultured alone and then washed away. Subsequently, SCC-12 cells were cultured and left to invade the matrix previously incubated with fibroblasts. Remarkably, SCC-12 cells invaded the matrix following the force-mediated tracks created by the stromal fibroblasts previously cultured, suggesting that physical conditioning of the supporting matrix and not some fibroblast-derived soluble factor was essential to lead invasion [72]. The authors of this study were able to show that absence of force-mediated remodeling of the matrix did not impair fibroblasts motility, but abolished invasion by SCC-12 cells. Several members of the integrin family, as well as MMPs and RhoGTPases (a family of small GTPases that generate contractile force), were essential both in leading invading fibroblasts and for matrix remodeling [72,73].

The congruence between the data derived from *in vitro* models and the data derived from gene-expression profiling studies regarding integrin signaling strongly suggests the relevance of these molecules in invasion and development of metastases [74]. As such, many therapeutic strategies directed against integrin signaling are under investigation [75,76].

## 3. Conclusions

HNSCC is a molecularly complex and heterogeneous disease. Such heterogeneity is also reflected in the mechanisms of invasion. Recent high-throughput profiling studies have unveiled the relevance of the mutational status of several key genes. Among the most prominent genes, *NOTCH1* has been suggested to be able to act both as a tumor-suppressor regulating cell squamous differentiation and as an oncogene promoting EMT. NGS studies have equally shown a high percentage of mutations of PI3K pathway members, pointing out a cardinal role in invasiveness and providing new perspectives for molecular targeted therapy. Additionally, the essential functions in invasiveness of several molecules involved in biological processes such as digestion and remodeling of the ECM, chemotaxis, antigen presentation, and angiogenesis have been confirmed in gene-profiling studies, providing an accurate picture of the invasive process in HNSCC.

The practical implementation of these findings in the field of clinical oncology still needs extensive validation.

## References

[1] Argiris A., Karamouzis M.V., Raben D., Ferris R.L. (2008). Head and neck cancer. Lancet.

[2] Jemal A., Siegel R., Xu J., Ward E. (2010). Cancer statistics, 2010. CA Cancer J. Clin..

[3] Goodwin W.J. (2000). Salvage surgery for patients with recurrent squamous cell carcinoma of the upper aerodigestive tract: When do the ends justify the means?. Laryngoscope.

[4] Bonner J.A., Harari P.M., Giralt J., Cohen R.B., Jones C.U., Sur R.K., Raben D., Baselga J., Spencer S.A., Zhu J. (2010). Radiotherapy plus cetuximab for locoregionally advanced head and neck cancer: 5-year survival data from a phase 3 randomised trial, and relation between cetuximab-induced rash and survival. Lancet Oncol..

[5] Chaturvedi A.K., Engels E.A., Pfeiffer R.M., Hernandez B.Y., Xiao W., Kim E., Jiang B., Goodman M.T., Sibug-Saber M., Cozen W. (2011). Human papillomavirus and rising oropharyngeal cancer incidence in the united states. J. Clin. Oncol..

[6] Marur S., D’Souza G., Westra W.H., Forastiere A.A. (2010). HPV-associated head and neck cancer: A virus-related cancer epidemic. Lancet Oncol..

[7] Ferlito A., Shaha A.R., Silver C.E., Rinaldo A., Mondin V. (2001). Incidence and sites of distant metastases from head and neck cancer. ORL J. Otorhinolaryngol. Relat. Spec..

[8] Leemans C.R., Braakhuis B.J., Brakenhoff R.H. (2011). The molecular biology of head and neck cancer. Nat. Rev. Cancer.

[9] Hornberg J.J., Bruggeman F.J., Westerhoff H.V., Lankelma J. (2006). Cancer: A systems biology disease. Bio. Syst..

[10] Bruggeman F.J., Westerhoff H.V. (2007). The nature of systems biology. Trends Microbiol..

[11] Stricker T., Catenacci D.V., Seiwert T.Y. (2011). Molecular profiling of cancer—The future of personalized cancer medicine: A primer on cancer biology and the tools necessary to bring molecular testing to the clinic. Semin. Oncol..

[12] Cancer Genome Atlas Network (2015). Comprehensive genomic characterization of head and neck squamous cell carcinomas. Nature.

[13] Seiwert T.Y., Zuo Z., Keck M.K., Khattri A., Pedamallu C.S., Stricker T., Brown C., Pugh T.J., Stojanov P., Cho J. (2015). Integrative and comparative genomic analysis of HPV-positive and HPV-negative head and neck squamous cell carcinomas. Clin. Cancer Res..

[14] Nichols A.C., Palma D.A., Chow W., Tan S., Rajakumar C., Rizzo G., Fung K., Kwan K., Wehrli B., Winquist E. (2013). High frequency of activating pik3ca mutations in human papillomavirus-positive oropharyngeal cancer. JAMA Otolaryngol. Head Neck Surg..

[15] Oganesyan G., Saha S.K., Guo B., He J.Q., Shahangian A., Zarnegar B., Perry A., Cheng G. (2006). Critical role of TRAF3 in the toll-like receptor-dependent and -independent antiviral response. Nature.

[16] Hayden M.S., Ghosh S. (2008). Shared principles in NF-kappab signaling. Cell.

[17] Sepiashvili L., Bruce J.P., Huang S.H., O’Sullivan B., Liu F.F., Kislinger T. (2015). Novel insights into head and neck cancer using next-generation “omic” technologies. Cancer Res..

[18] Lin Y., Bai L., Chen W., Xu S. (2010). The NF-kappab activation pathways, emerging molecular targets for cancer prevention and therapy. Expert Opin. Ther. Targets.

[19] Smith A., Teknos T.N., Pan Q. (2013). Epithelial to mesenchymal transition in head and neck squamous cell carcinoma. Oral Oncol..

[20] Chen C., Zimmermann M., Tinhofer I., Kaufmann A.M., Albers A.E. (2013). Epithelial-to-mesenchymal transition and cancer stem(-like) cells in head and neck squamous cell carcinoma. Cancer Lett..

[21] Hanahan D., Weinberg R.A. (2011). Hallmarks of cancer: The next generation. Cell.

[22] Kalluri R., Weinberg R.A. (2009). The basics of epithelial-mesenchymal transition. J. Clin. Investig..

[23] Thiery J.P. (2002). Epithelial-mesenchymal transitions in tumour progression. Nat. Rev. Cancer.

[24] Scheel C., Weinberg R.A. (2012). Cancer stem cells and epithelial-mesenchymal transition: Concepts and molecular links. Semin. Cancer Biol..

[25] Chung C.H., Parker J.S., Karaca G., Wu J., Funkhouser W.K., Moore D., Butterfoss D., Xiang D., Zanation A., Yin X. (2004). Molecular classification of head and neck squamous cell carcinomas using patterns of gene expression. Cancer Cell.

[26] Chung C.H., Parker J.S., Ely K., Carter J., Yi Y., Murphy B.A., Ang K.K., El-Naggar A.K., Zanation A.M., Cmelak A.J. (2006). Gene expression profiles identify epithelial-to-mesenchymal transition and activation of nuclear factor-kappab signaling as characteristics of a high-risk head and neck squamous cell carcinoma. Cancer Res..

[27] Ginos M.A., Page G.P., Michalowicz B.S., Patel K.J., Volker S.E., Pambuccian S.E., Ondrey F.G., Adams G.L., Gaffney P.M. (2004). Identification of a gene expression signature associated with recurrent disease in squamous cell carcinoma of the head and neck. Cancer Res..

[28] Yan B., Yang X., Lee T.L., Friedman J., Tang J., van Waes C., Chen Z. (2007). Genome-wide identification of novel expression signatures reveal distinct patterns and prevalence of binding motifs for p53, nuclear factor-kappab and other signal transcription factors in head and neck squamous cell carcinoma. Genome Biol..

[29] De Cecco L., Bossi P., Locati L., Canevari S., Licitra L. (2014). Comprehensive gene expression meta-analysis of head and neck squamous cell carcinoma microarray data defines a robust survival predictor. Ann. Oncol..

[30] Stransky N., Egloff A.M., Tward A.D., Kostic A.D., Cibulskis K., Sivachenko A., Kryukov G.V., Lawrence M.S., Sougnez C., McKenna A. (2011). The mutational landscape of head and neck squamous cell carcinoma. Science.

[31] Agrawal N., Frederick M.J., Pickering C.R., Bettegowda C., Chang K., Li R.J., Fakhry C., Xie T.X., Zhang J., Wang J. (2011). Exome sequencing of head and neck squamous cell carcinoma reveals inactivating mutations in Notch1. Science.

[32] Miele L., Golde T., Osborne B. (2006). Notch signaling in cancer. Curr. Mol. Med..

[33] Koch U., Radtke F. (2010). Notch signaling in solid tumors. Curr. Top. Dev. Biol..

[34] Hu Y.Y., Zheng M.H., Zhang R., Liang Y.M., Han H. (2012). Notch signaling pathway and cancer metastasis. Adv. Exp. Med. Biol..

[35] Garcia A., Kandel J.J. (2012). Notch: A key regulator of tumor angiogenesis and metastasis. Histol. Histopathol..

[36] Bailey J.M., Singh P.K., Hollingsworth M.A. (2007). Cancer metastasis facilitated by developmental pathways: Sonic hedgehog, Notch, and bone morphogenic proteins. J. Cell. Biochem..

[37] South A.P., Cho R.J., Aster J.C. (2012). The double-edged sword of Notch signaling in cancer. Semin. Cell Dev. Biol..

[38] Extance A. (2010). Alzheimer’s failure raises questions about disease-modifying strategies. Nat. Rev. Drug Discov..

[39] Sun W., Gaykalova D.A., Ochs M.F., Mambo E., Arnaoutakis D., Liu Y., Loyo M., Agrawal N., Howard J., Li R. (2014). Activation of the Notch pathway in head and neck cancer. Cancer Res..

[40] Hayward P., Kalmar T., Arias A.M. (2008). Wnt/Notch signalling and information processing during development. Development.

[41] Li Y., Ma J., Qian X., Wu Q., Xia J., Miele L., Sarkar F.H., Wang Z. (2013). Regulation of emt by Notch signaling pathway in tumor progression. Curr. Cancer Drug Targets.

[42] Wang N.J., Sanborn Z., Arnett K.L., Bayston L.J., Liao W., Proby C.M., Leigh I.M., Collisson E.A., Gordon P.B., Jakkula L. (2011). Loss-of-function mutations in Notch receptors in cutaneous and lung squamous cell carcinoma. Proc. Natl. Acad. Sci. USA.

[43] Dorsam R.T., Gutkind J.S. (2007). G-protein-coupled receptors and cancer. Nat. Rev. Cancer.

[44] McCubrey J.A., Steelman L.S., Abrams S.L., Lee J.T., Chang F., Bertrand F.E., Navolanic P.M., Terrian D.M., Franklin R.A., D’Assoro A.B. (2006). Roles of the RAF/MEK/ERK and PI3K/PTEN/AKT pathways in malignant transformation and drug resistance. Adv. Enzyme Regul..

[45] Meyer S.C., Levine R.L. (2014). Molecular pathways: Molecular basis for sensitivity and resistance to JAK kinase inhibitors. Clin. Cancer Res..

[46] Lui V.W., Hedberg M.L., Li H., Vangara B.S., Pendleton K., Zeng Y., Lu Y., Zhang Q., Du Y., Gilbert B.R. (2013). Frequent mutation of the PI3K pathway in head and neck cancer defines predictive biomarkers. Cancer Discov..

[47] Fruman D.A., Rommel C. (2014). PI3K and cancer: Lessons, challenges and opportunities. Nat. Rev. Drug Discov..

[48] Gonzalez-Angulo A.M., Ferrer-Lozano J., Stemke-Hale K., Sahin A., Liu S., Barrera J.A., Burgues O., Lluch A.M., Chen H., Hortobagyi G.N. (2011). PI3K pathway mutations and PTEN levels in primary and metastatic breast cancer. Mol. Cancer Ther..

[49] Daneshmand M., Hanson J.E., Nabavi M., Hilton J.F., Vandermeer L., Kanji F., Dent S.F., Clemons M., Lorimer I.A. (2012). Detection of pik3ca mutations in breast cancer bone metastases. ISRN Oncol..

[50] Yuan T.L., Cantley L.C. (2008). PI3K pathway alterations in cancer: Variations on a theme. Oncogene.

[51] Psyrri A., Seiwert T.Y., Jimeno A. (2013). Molecular pathways in head and neck cancer: EGFR, PI3K, and more. Am. Soc. Clin. Oncol. Educ. Book.

[52] Engelman J.A. (2009). Targeting PI3K signalling in cancer: Opportunities, challenges and limitations. Nat. Rev. Cancer.

[53] Yu Y.H., Kuo H.K., Chang K.W. (2008). The evolving transcriptome of head and neck squamous cell carcinoma: A systematic review. PLoS ONE.

[54] Waugh D.J., Wilson C. (2008). The interleukin-8 pathway in cancer. Clin. Cancer Res..

[55] Takeuchi K., Ito F. (2011). Receptor tyrosine kinases and targeted cancer therapeutics. Biol. Pharm. Bull..

[56] Nisa L., Aebersold D.M., Giger R., Zimmer Y., Medova M. (2014). Biological, diagnostic and therapeutic relevance of the met receptor signaling in head and neck cancer. Pharmacol. Ther..

[57] Maiti G.P., Mondal P., Mukherjee N., Ghosh A., Ghosh S., Dey S., Chakrabarty J., Roy A., Biswas J., Roychoudhury S. (2013). Overexpression of egfr in head and neck squamous cell carcinoma is associated with inactivation of SH3Gl2 and CDC25A genes. PLoS ONE.

[58] Radhakrishnan R., Solomon M., Satyamoorthy K., Martin L.E., Lingen M.W. (2008). Tissue microarray—A high-throughput molecular analysis in head and neck cancer. J. Oral Pathol. Med..

[59] Kessenbrock K., Plaks V., Werb Z. (2010). Matrix metalloproteinases: Regulators of the tumor microenvironment. Cell.

[60] Hua H., Li M., Luo T., Yin Y., Jiang Y. (2011). Matrix metalloproteinases in tumorigenesis: An evolving paradigm. Cell. Mol. Life Sci..

[61] Cai K.Q., Yang W.L., Capo-Chichi C.D., Vanderveer L., Wu H., Godwin A.K., Xu X.X. (2007). Prominent expression of metalloproteinases in early stages of ovarian tumorigenesis. Mol. Carcinog..

[62] Di Carlo A. (2013). Evaluation of neutrophil gelatinase-associated lipocalin (NGAL), matrix metalloproteinase-9 (MMP-9) and their complex MMP-9/NGAL in sera and urine of patients with kidney tumors. Oncol. Lett..

[63] Bouchet S., Bauvois B. (2014). Neutrophil gelatinase-associated lipocalin (NGAL), pro-matrix metalloproteinase-9 (pro-MMP-9) and their complex pro-MMP-9/NGAL in leukaemias. Cancers.

[64] Schreiber R.D., Old L.J., Smyth M.J. (2011). Cancer immunoediting: Integrating immunity’s roles in cancer suppression and promotion. Science.

[65] Ye H., Yu T., Temam S., Ziober B.L., Wang J., Schwartz J.L., Mao L., Wong D.T., Zhou X. (2008). Transcriptomic dissection of tongue squamous cell carcinoma. BMC Genomics.

[66] Nagata M., Fujita H., Ida H., Hoshina H., Inoue T., Seki Y., Ohnishi M., Ohyama T., Shingaki S., Kaji M. (2003). Identification of potential biomarkers of lymph node metastasis in oral squamous cell carcinoma by cDNA microarray analysis. Int. J. Cancer.

[67] Kainuma K., Katsuno S., Hashimoto S., Oguchi T., Suzuki N., Asamura K., Usami S. (2006). Differences in the expression of genes between normal tissue and squamous cell carcinomas of head and neck using cancer-related gene cDNA microarray. Acta Otolaryngol..

[68] Kondoh N., Ishikawa T., Ohkura S., Arai M., Hada A., Yamazaki Y., Kitagawa Y., Shindoh M., Takahashi M., Ando T. (2008). Gene expression signatures that classify the mode of invasion of primary oral squamous cell carcinomas. Mol. Carcinog..

[69] Choi P., Chen C. (2005). Genetic expression profiles and biologic pathway alterations in head and neck squamous cell carcinoma. Cancer.

[70] Gottschlich S., Ambrosch P., Cordes C., Gorogh T., Schreiber S., Hasler R. (2006). Gene expression profiling of head and neck squamous cell carcinoma using cDNA microarrays. Int. J. Oncol..

[71] Zaidel-Bar R., Itzkovitz S., Ma’ayan A., Iyengar R., Geiger B. (2007). Functional atlas of the integrin adhesome. Nat. Cell Biol..

[72] Gaggioli C., Hooper S., Hidalgo-Carcedo C., Grosse R., Marshall J.F., Harrington K., Sahai E. (2007). Fibroblast-led collective invasion of carcinoma cells with differing roles for rhogtpases in leading and following cells. Nat. Cell Biol..

[73] Li H., Peyrollier K., Kilic G., Brakebusch C. (2014). Rho GTPases and cancer. BioFactors.

[74] Lathia J.D., Chigurupati S., Thundyil J., Selvaraj P.K., Mughal M.R., Woodruff T.M., Chan S.L., Karamyan V.T., Mattson M.P., Arumugam T.V. (2010). Pivotal role for beta-1 integrin in neurovascular remodelling after ischemic stroke. Exp. Neurol..

[75] Seguin L., Desgrosellier J.S., Weis S.M., Cheresh D.A. (2015). Integrins and cancer: Regulators of cancer stemness, metastasis, and drug resistance. Trends Cell Biol..

[76] Desgrosellier J.S., Cheresh D.A. (2010). Integrins in cancer: Biological implications and therapeutic opportunities. Nat. Rev. Cancer.

